# Intestinal Suture with the Use of a Potato Cylinder

**Published:** 1899-12

**Authors:** M. B. Saunders

**Affiliations:** Beaumont, Texas


For Texas Medical Journal.

### Intestinal Suture With the Use of a Potato Cylinder.

BY M. B. SAUNDERS, M. D., BEAUMONT, TEXAS.

I have never heard of the potato cylinder being used, but to my mind it has its advantages as well as its disadvantages over the inflated rubber cylinders. And in my hands, having used it on dogs, I see no reason why it should not be an efficient substitute for the latter.

The cylinder is cut from an Irish potato; the size to be the diameter of the gut, and about an inch long, and the walls just thick enough to give sufficient body to it. This is then put in a solution of salt in water, which slightly hardens it, where it remains until ready for use.

The gut is then brought out and prepared in the usual way, stripping back the contents of the bowel and clamping, so as to prevent soiling the field of operation. The intestine is then cut straight across with scissors, and the lumen is cleansed of any débris that may be found. The cylinder is introduced for half its length into one end and then telescoped into the other end, care being taken to see that the ends of the intestine are in correct apposition. Now, by means of two rows of sutures they are united. The first sutures go through all the layers of the gut and are inserted quite close to the margin; care being taken to see that the thick layers of the muscularis are brought into intimate apposition. We may use for this the interrupted or continuous suture; I prefer the continuous, buttonhole or blanket suture, as it is just as good, and more rapid work may be accomplished.

The second row are Lembert sutures, and are intended to bring the serous coats together; and in doing this they must be taken further back; so as to imbed the first row of sutures, and it is advisable to include with the serous layer a portion of the muscularis layer, and in this way the serous folds are made firmer, and can be brought into firmer apposition. Here I give preference to the interrupted mattress or inverted mattress suture.

Having knotted these sutures they should be cut quite close; then there remains for us to remove our clamps. The intestinal loop at once fills with gas and the cylinder, which of course is hollow, is to be squeezed further down the gut, so as to prevent interfering with the healing process.

The greatest advantages are: bringing all the layers of the bowel in accurate apposition; the readiness with which the cylinder may be obtained, and there being no danger of puncturing it in making the through sutures, and the ability to remove it from the field of operation.

